# Brick Walls and Broken Hearts: Physicians Draw how they Feel About Treating Pain and Addiction

**DOI:** 10.1007/s11606-024-09205-8

**Published:** 2024-12-10

**Authors:** Lisa R. Villarroel, Aram S. Mardian, Cynthia O. Townsend, Steven R. Brown

**Affiliations:** 1 Public Health Services, Arizona Department of Health Services, Phoenix, USA; 2 Chronic Pain Wellness Center, Phoenix Veterans Affairs Health Care System, Phoenix, USA; 3 Department of Psychiatry and Psychology, Mayo Clinic, Phoenix, USA; 4 Department of Anesthesiology, Pain Rehabilitation Center, Mayo Clinic, Phoenix, USA; 5 Department of Family, Community and Preventive Medicine, University of Arizona College of Medicine–Phoenix, Phoenix, USA

**Keywords:** pain, addiction, stigma, physician drawing, primary care, bias

## Abstract

As part of a continuing medical education activity, primary care physicians in Arizona were asked to draw how they feel about treating patients with chronic pain and addiction. Their drawings, complete with cliffs, walls, torn-out hair, and connected hearts, make for a harrowing look at burnout, angst, and empathy among physicians who treat this subset of patients. Public health and exercise facilitators were troubled, leading them to publish this essay along with a selection of the drawings that depict the feelings of the physicians who feel ill-prepared to treat this patient population, which is only growing in number and needs.

**Supplementary Information:**

The online version contains supplementary material available at 10.1007/s11606-024-09205-8.

“But what if I don’t know how to draw?”

At a continuing medical education event dedicated to primary care physicians who treat chronic pain and addiction, the facilitator answered, “It doesn’t need to be perfect. Use the markers to draw how you *feel* about treating patients with pain.”

It was the eve of the COVID-19 pandemic. Addiction medicine specialists and public health officials who sponsored the event stood together, watching a room of seventy physicians draw in permanent marker on cards labeled PAIN. After a few minutes, someone whisked the drawings away, putting new cards in their place.

“Now,” said the facilitator, “Draw how you feel about treating patients with *addiction*.”

And that was it.

The conference went on. Through round tables, presentations, and group work, attendees discussed case studies, opioid tapering practices, and the stigma that affects patients with pain and addiction. The programming encouraged providers to treat the whole person and prescribe medications for opioid use disorder.

Nearing the end, there was an agenda item marked “Reflection.” The facilitator who had given the drawing instructions took the lectern and said, “Let’s look at how some of us in the room feel about treating patients with pain.”

The facilitator, a nationally recognized psychologist on pain and addiction, described what she saw on this anonymized selection of drawings projected on the screen. She detailed faces with zigzag mouths, tears flowing, stick figures running off cliffs or into brick walls, doctors with grit teeth and burning hair. There were a few written phrases like “FRUSTRATED,” “Time is too much!!” and, pleadingly, “I’m here to help.” (Fig. 1).

**Figure 1:**
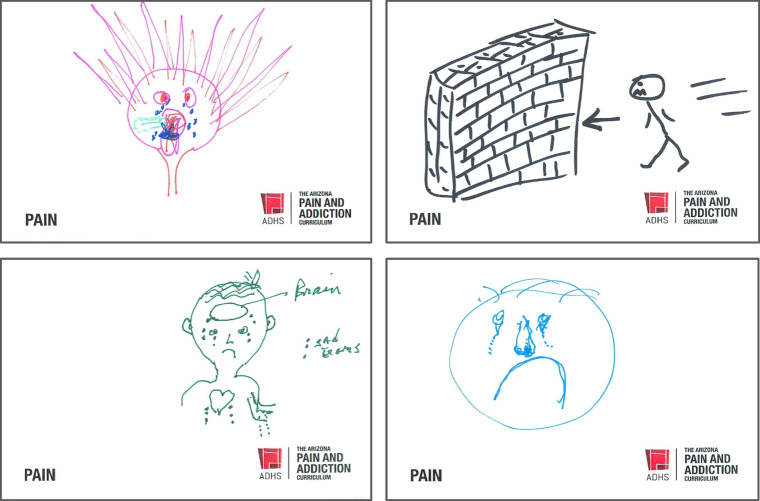
Selected drawings by physicians, who followed instructions to draw how they feel about treating patients with pain.

The room went very still.

“Let’s look now,” said the facilitator, “at how some of us in the room feel about treating patients with *addiction*.”

The facilitator projected the drawings: figures separated by walls, hearts broken with jagged lines. There were signs of connection in figures that reached out or were joined with dotted lines, but there was also self-flagellation, slamming doors, and words like “hopeless,” or “alone and inadequate” scrawled next to unhappy faces (Fig. 2).

**Figure 2:**
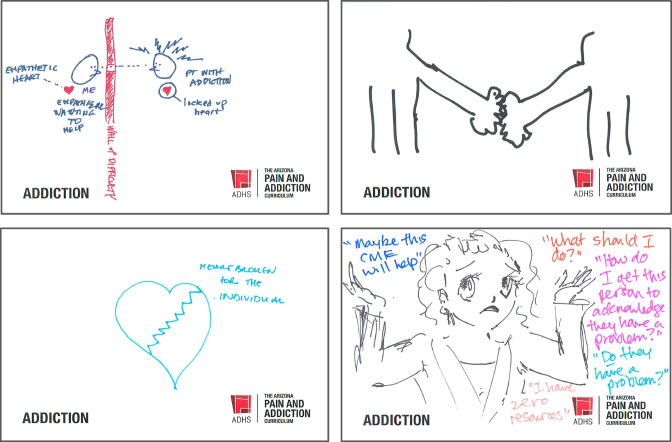
Selected drawings by physicians, who followed instructions to draw how they feel about treating patients with addiction.

In post-conference evaluations, attendees revealed that the majority of them would recommend the drawing exercise as a reflection practice for physicians at continuing medical education events.

In post-conference discussions, the facilitators were circumspect, reflecting on the signs of burnout and angst seen in the drawings. One of the support staff hissed into a public health official’s ear, “My sister died from an overdose. This is how doctors felt about treating her?”

The question was what to do with these findings. This exercise was not research. It was not blinded. There weren’t controls over marker colors. No one interviewed the physicians to clarify their drawings. There are no phenomenological criteria we wish to retroactively engineer.

To us, these are raw glimpses of a fraught population—who themselves are treating a fraught population—that must be shared. As public health crises of chronic pain and addiction in the United States^1^ drive an increasing number of patients to seek care in a primary care clinic, it seems important that physicians feel just as heartbroken and frustrated as their patients.

Was there a thematic difference between the pain and addiction drawings? More disgust in the pain drawings? More hearts breaking or bleeding in the addiction drawings? Has addiction, long considered a “moral weakness” or a negative health behavior, finally been recognized as a medical condition? Are the heightened emotions a reflection of physicians’ compassion fatigue, overwork, or both?

A few attempts have been made to answer these questions, albeit in prose. Conventional research cannot capture the emotional complexity of physicians’ experience in the clinic, but studies suggest high rates of burnout^2^ and feelings that these patients are time-consuming, difficult, and frustrating to care for.^3,4^ A table in a recent systematic review^5^ details thirty-five reasons for physician reluctance to intervene in treating patients evidencing addiction. Reading between the lines, physicians appear utterly overwhelmed.

The problem is not specific to the personalities or capacities of individual providers. It cannot be solved by a wellness retreat, addiction medicine training, or an ICD-10 code. Primary care as a whole needs more reimbursement. Patients with chronic health conditions and their families need more support. Physicians need more resources and time to care for a population that has fallen through the cracks of our healthcare system.

Maybe markers and paper can nudge us toward system change along with survey tables and bar charts (see Appendices 1 and 2). In their raw and visceral expression, drawings like these offer a glimpse into the inner lives of physicians as morbidity and mortality from chronic pain and addiction increase in Arizona and the nation at large.

To physicians who treat patients with pain and addiction: Share these drawings and add your voices to them. Come together to lower the brick walls drawn by your colleagues. Champion the need for whole person care, longer time with complex patients, and reimbursement that matches your care.

And to the staff member whose sister died from an overdose? Look to the hearts in the drawings. There are hearts everywhere.

## Supplementary Information

Below is the link to the electronic supplementary material.

Supplementary file1 (Additional pain drawings) (PDF 1761 KB)

Supplementary file2 (Additional addiction drawings) (PDF 1536 KB)
