# Supplementary material for: Brick Walls and Broken Hearts: Physicians Draw how they Feel About Treating Pain and Addiction
Source: J Gen Intern Med. 2024 Dec 10;40(5):1194–6. doi: 10.1007/s11606-024-09205-8 (PMC11968578; doi:10.1007/s11606-024-09205-8)

APPENDIX A: Selected drawings by physicians, who followed instructions to draw how they feel about treating patients with pain.

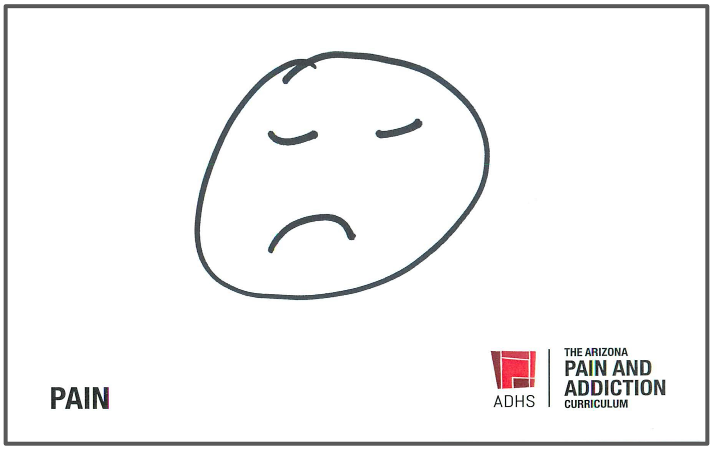

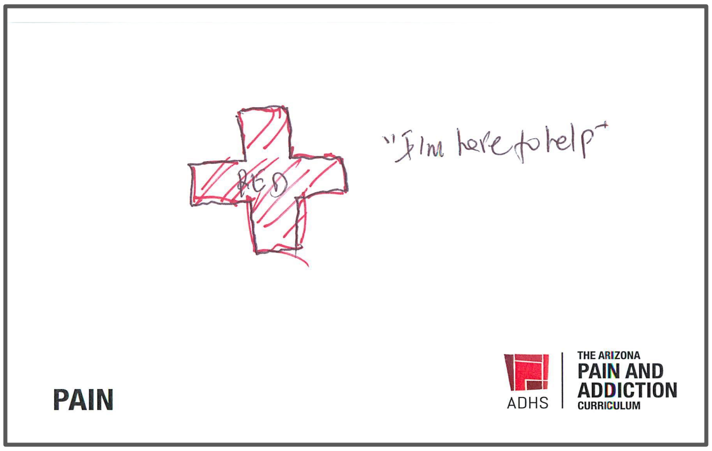

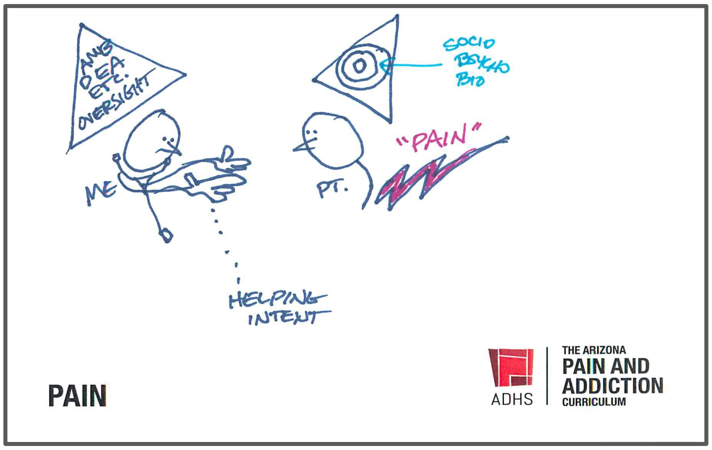

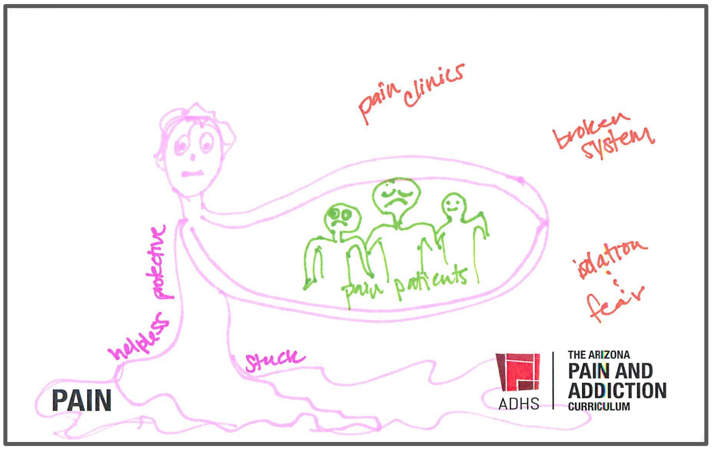

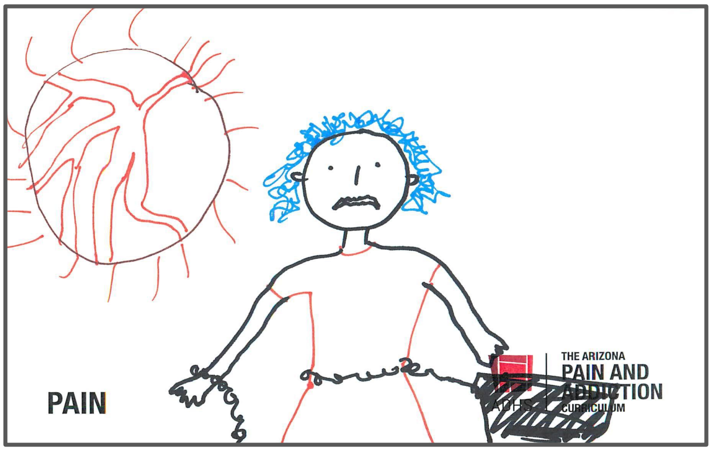

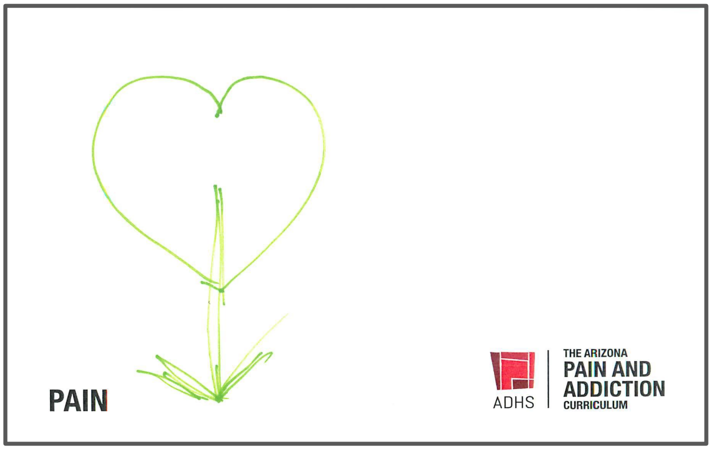

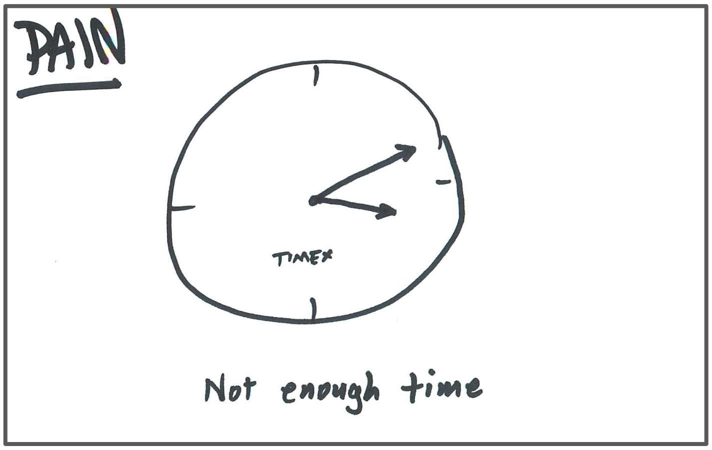

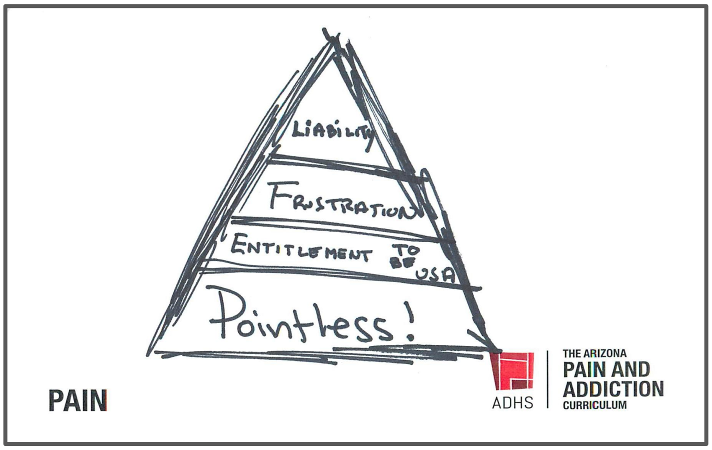

APPENDIX A (page 2): Selected drawings by physicians, who followed instructions to draw how they feel about treating patients with pain.

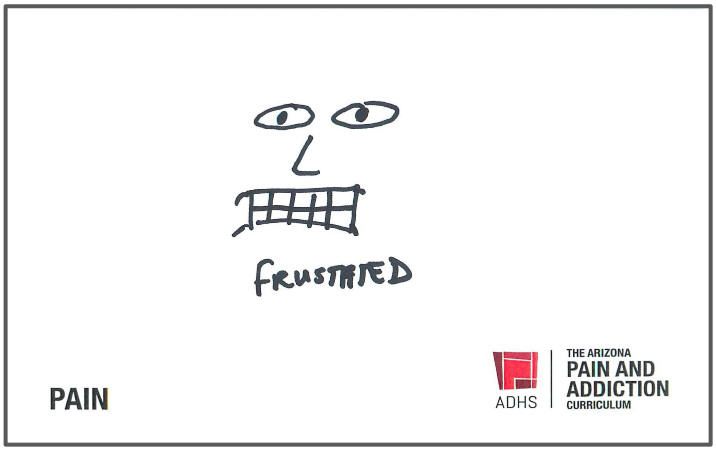

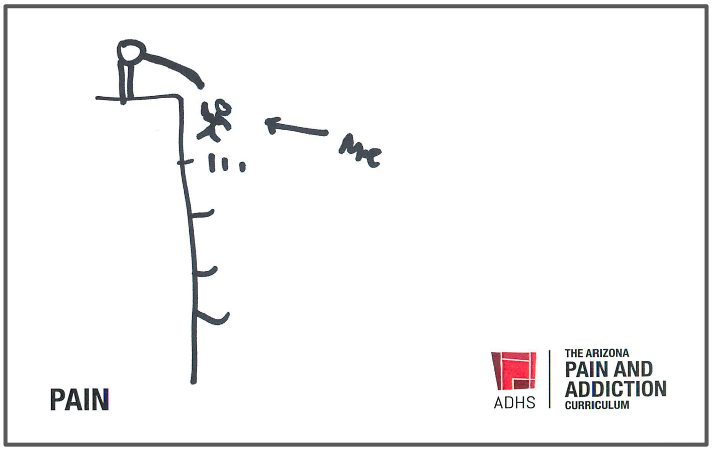

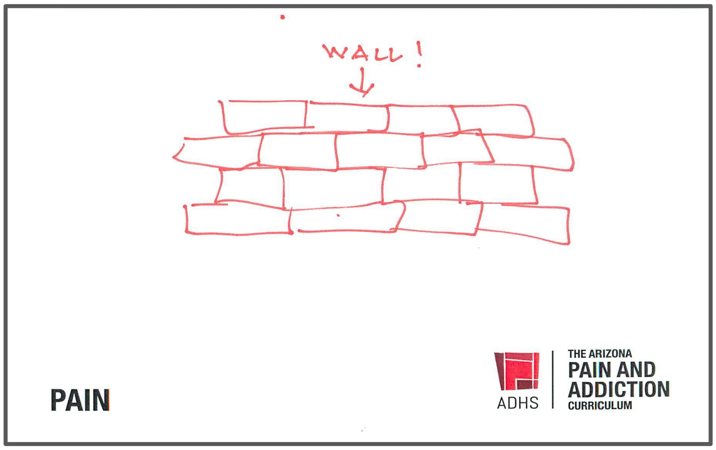

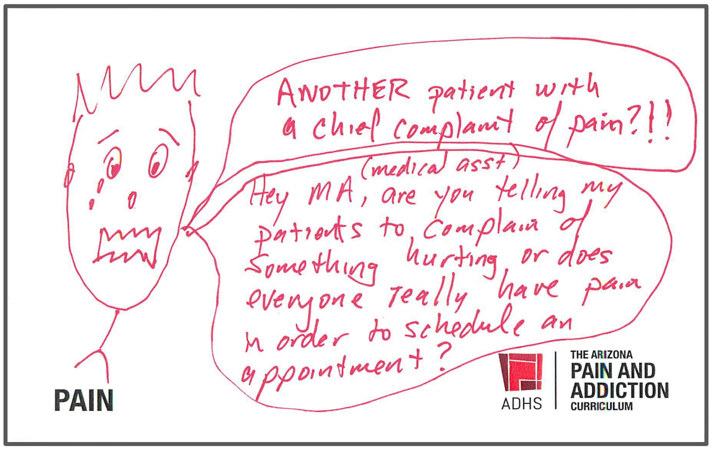

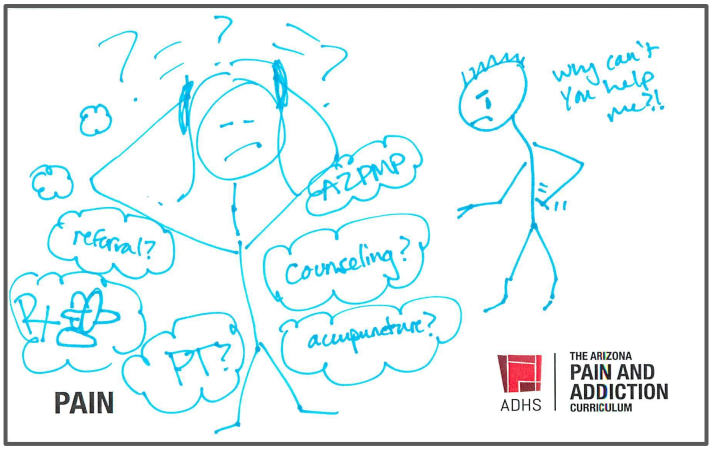

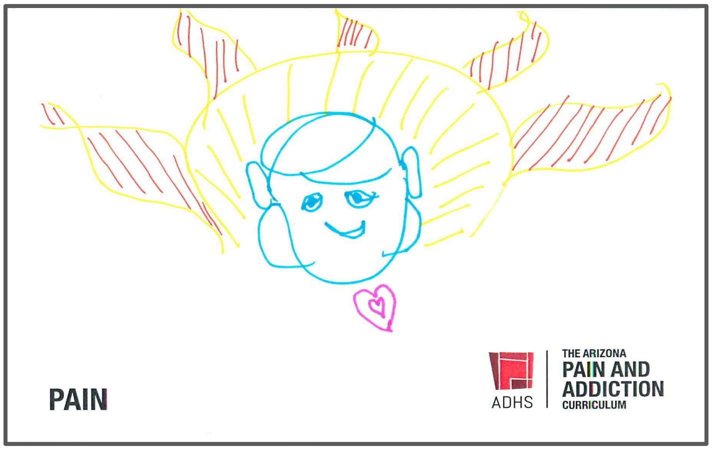

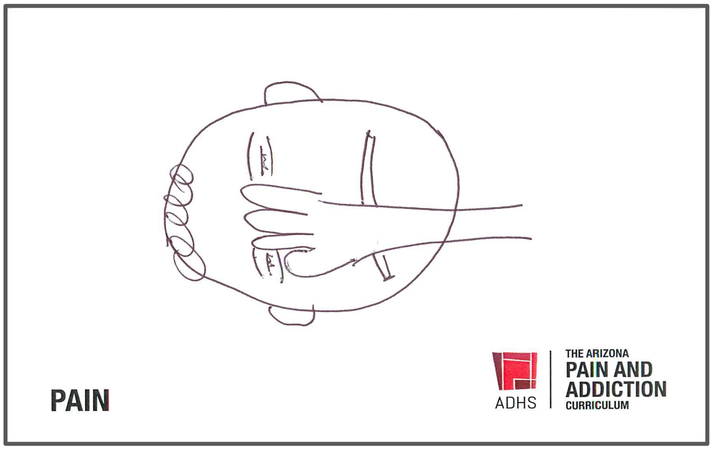

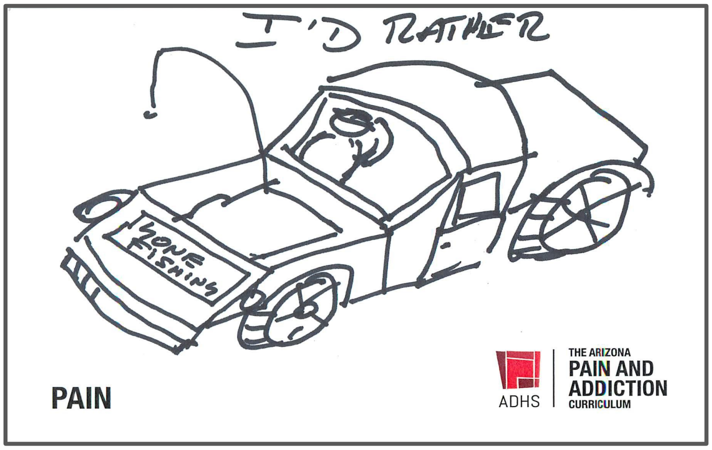

Supplement: Supplementary file 1 — Supplementary file1 (Additional pain drawings) (PDF 1761 KB) [file 11606_2024_9205_MOESM1_ESM.pdf]
