# Supplementary material for: Brick Walls and Broken Hearts: Physicians Draw how they Feel About Treating Pain and Addiction
Source: J Gen Intern Med. 2024 Dec 10;40(5):1194–6. doi: 10.1007/s11606-024-09205-8 (PMC11968578; doi:10.1007/s11606-024-09205-8)

APPENDIX B: Selected drawings by physicians, who followed instructions to draw how they feel about treating patients with addiction.

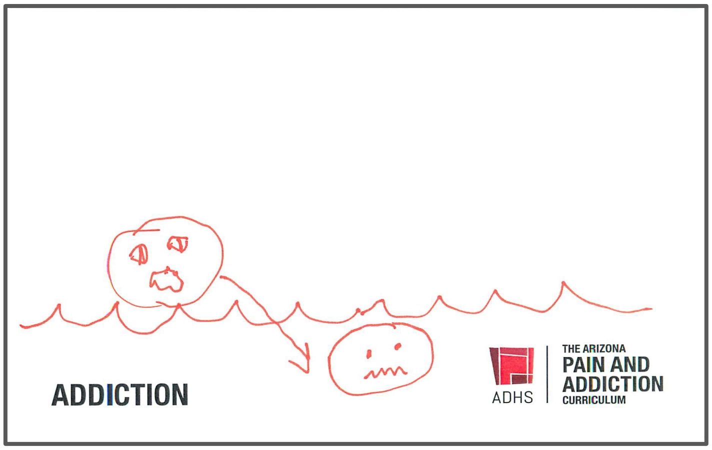

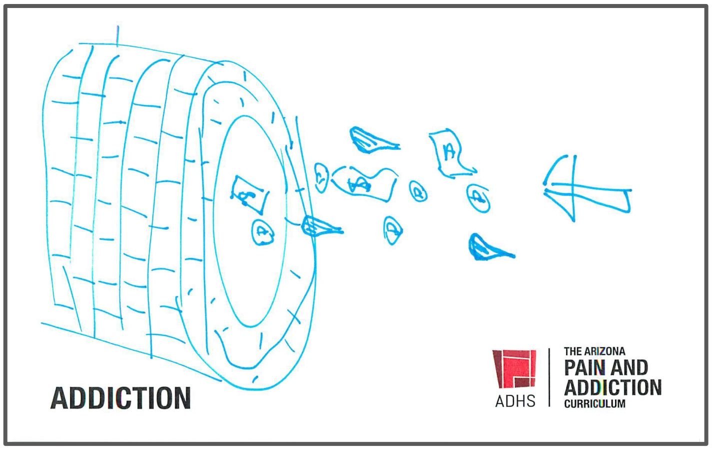

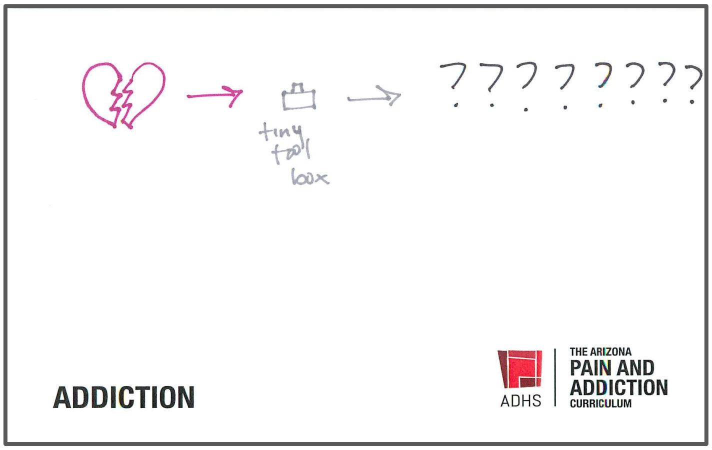

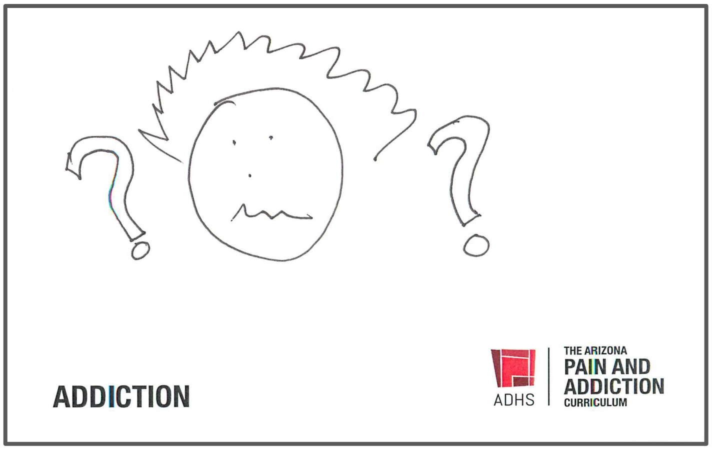

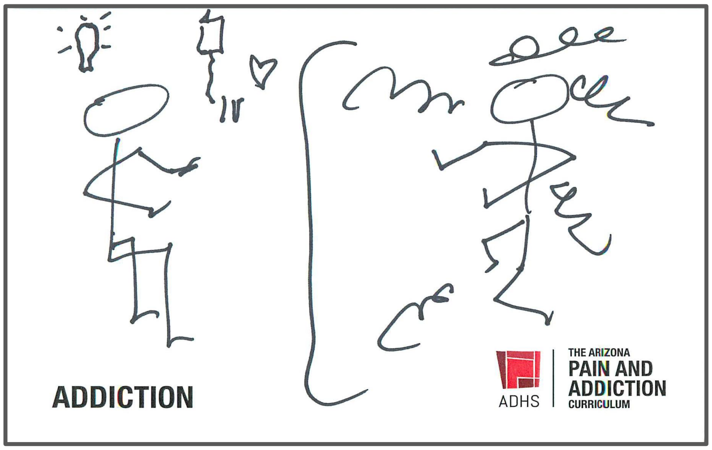

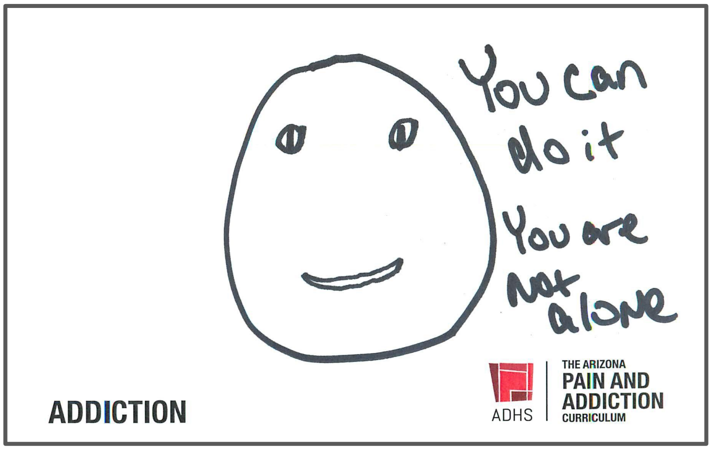

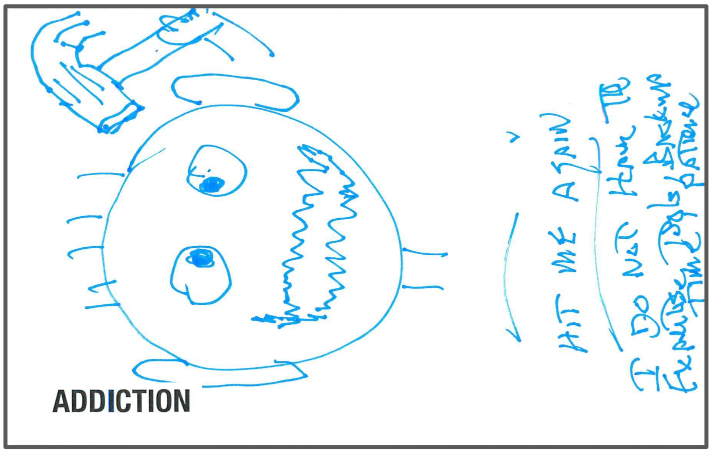

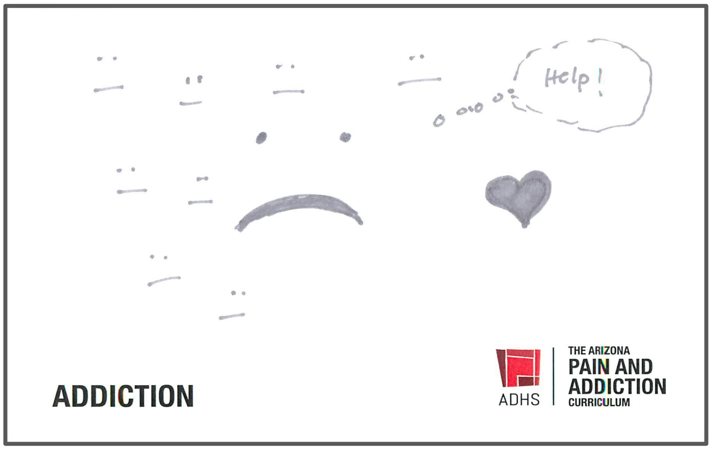

APPENDIX B (page 2): Selected drawings by physicians, who followed instructions to draw how they feel about treating patients with addiction.

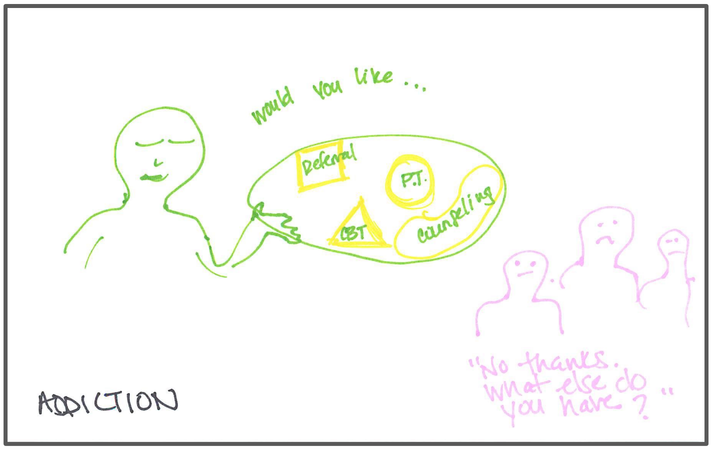

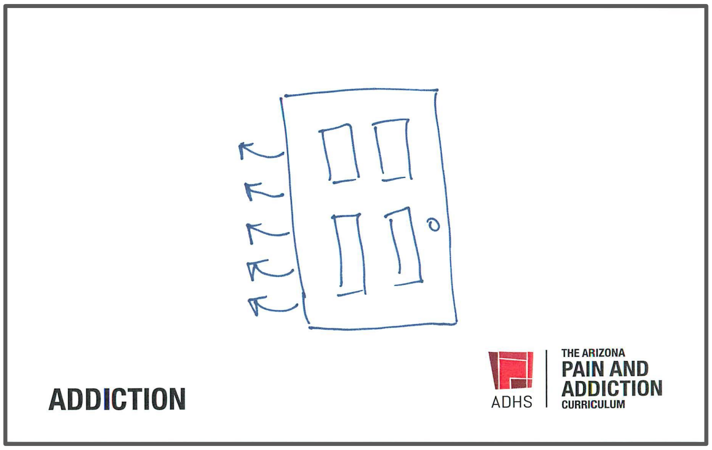

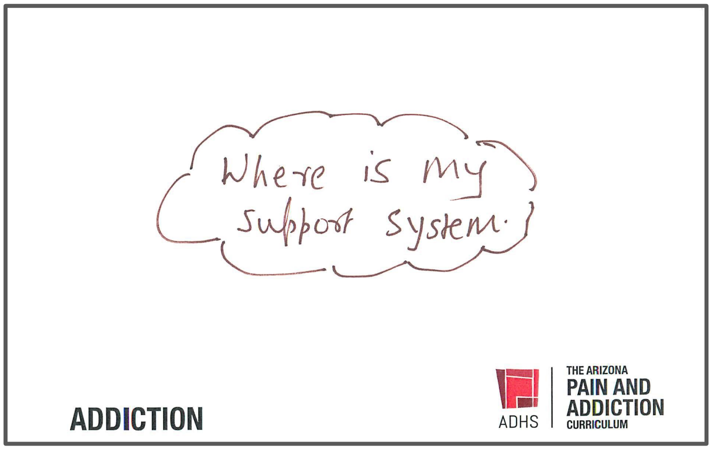

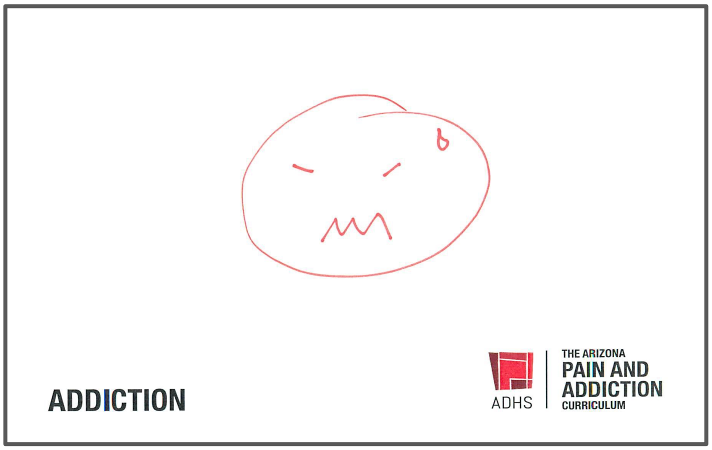

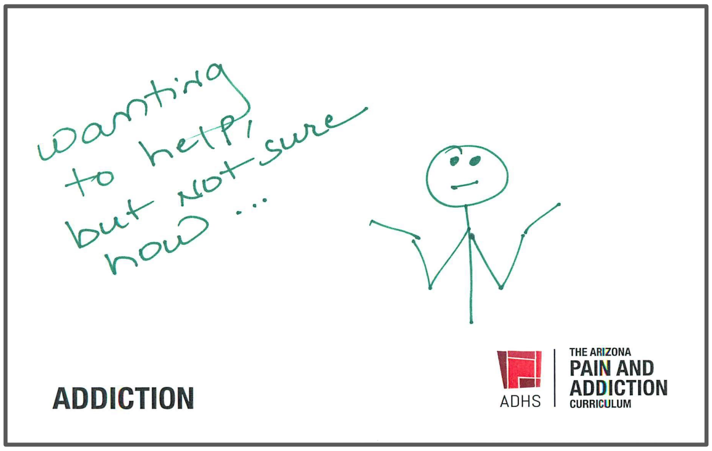

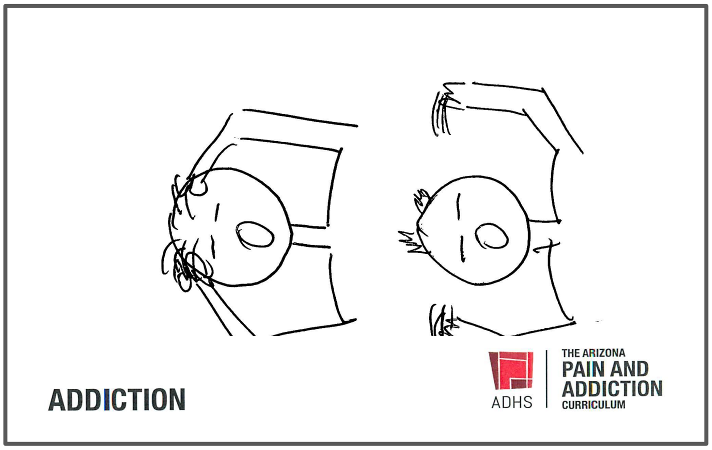

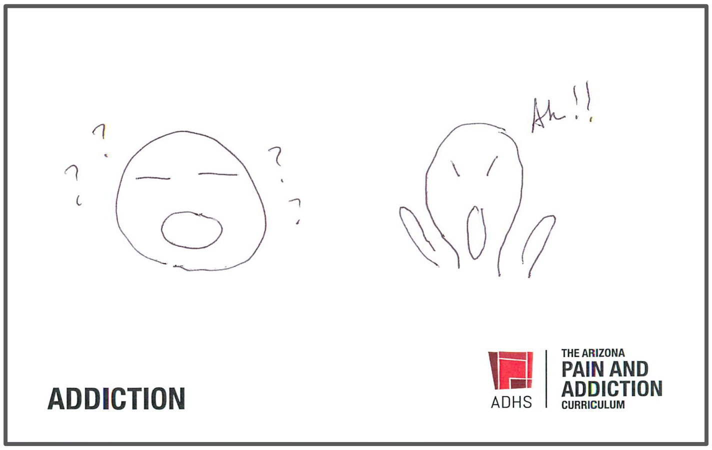

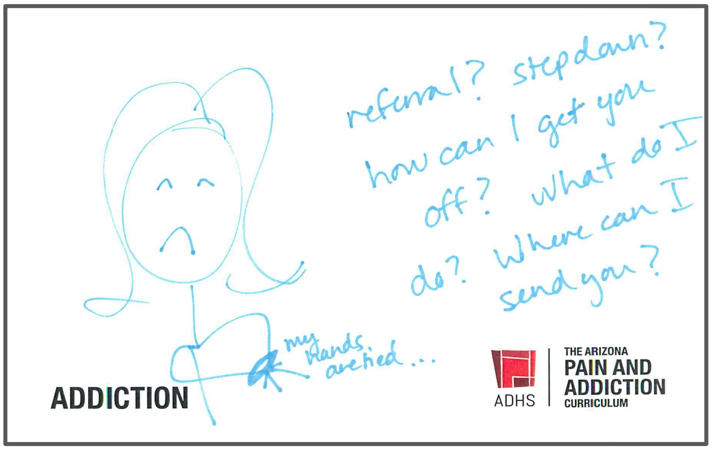

Supplement: Supplementary file 2 — Supplementary file2 (Additional addiction drawings) (PDF 1536 KB) [file 11606_2024_9205_MOESM2_ESM.pdf]
